# The Superior Function of the Subplate in Early Neocortical Development

**DOI:** 10.3389/fnana.2018.00097

**Published:** 2018-11-14

**Authors:** Heiko J. Luhmann, Sergei Kirischuk, Werner Kilb

**Affiliations:** Institute of Physiology, University Medical Center of the Johannes Gutenberg University Mainz, Mainz, Germany

**Keywords:** neocortex, development, subplate, connectivity, plasticity, pathology, review

## Abstract

During early development the structure and function of the cerebral cortex is critically organized by subplate neurons (SPNs), a mostly transient population of glutamatergic and GABAergic neurons located below the cortical plate. At the molecular and morphological level SPNs represent a rather diverse population of cells expressing a variety of genetic markers and revealing different axonal-dendritic morphologies. Electrophysiologically SPNs are characterized by their rather mature intrinsic membrane properties and firing patterns. They are connected via electrical and chemical synapses to local and remote neurons, e.g., thalamic relay neurons forming the first thalamocortical input to the cerebral cortex. Therefore SPNs are robustly activated at pre- and perinatal stages by the sensory periphery. Although SPNs play pivotal roles in early neocortical activity, development and plasticity, they mostly disappear by programmed cell death during further maturation. On the one hand, SPNs may be selectively vulnerable to hypoxia-ischemia contributing to brain damage, on the other hand there is some evidence that enhanced survival rates or alterations in SPN distribution may contribute to the etiology of neurological or psychiatric disorders. This review aims to give a comprehensive and up-to-date overview on the many functions of SPNs during early physiological and pathophysiological development of the cerebral cortex.

## Introduction

**FIGURE 1:**
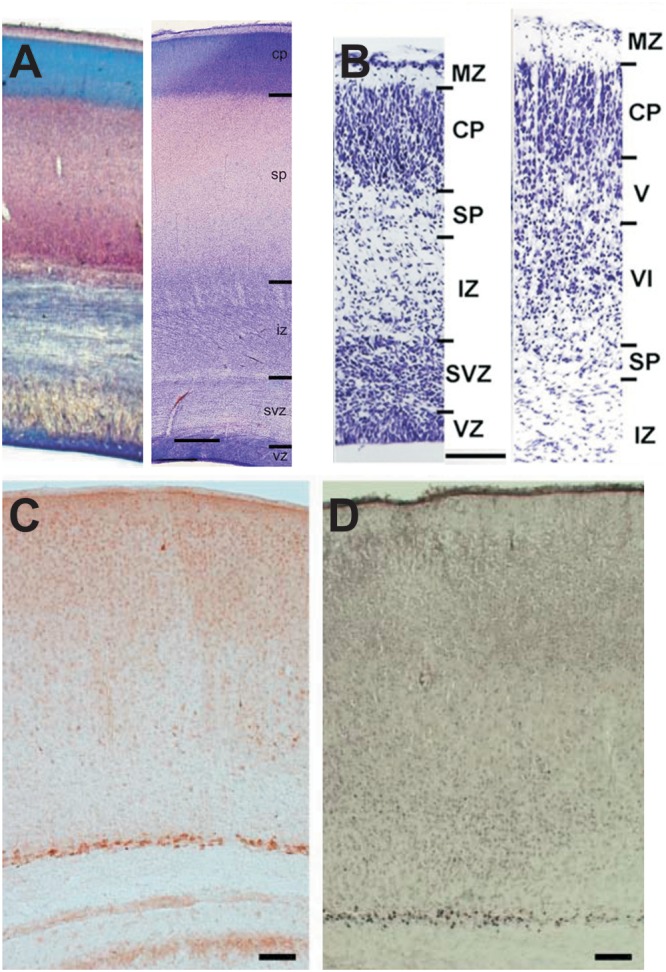
Appearance of the SP in developing human and mouse cerebral cortex. **(A)** Cresylviolet (left) and Nissl (right) stained sections from human fetal cortex at postconceptional weeks 18 and 22–24, respectively (modified with permission from Kostovic et al. (2002) and Judas et al. (2013). Note that the thickness of the SP exceeds that of the CP. **(B)** Nissl stained sections of the mouse cortex at E18.5 and P1 (modified with permission from Hayano et al., 2014). Note that SPNs represent a small band of neurons below the cortical plate/layer 6. **(C)** Immunhistochemical processed section of a P8 mouse cortex illustrating that Cplx3-positive Neurons are located exclusively in the SP (with permission from Hoerder-Suabedissen et al., 2009). **(D)** Nurr1 antibodies also stains exclusively SPNs (with permission from Hoerder-Suabedissen et al., 2009). Scale bars 1 mm in **(A)** and 100 μm in **(B–D)**. MZ, marginal zone; CP, cortical plate; SP, subplate; IZ, intermediate zone; SVZ, subventricular zone; VZ, ventricular zone; V and VI, layer V and VI, respectively.

The subplate (SP) was first described in the human cerebral cortex (Kostovic and Molliver, 1974), 3 years later in the fetal macaque (Rakic, 1977) and in rats (Rickmann et al., 1977), and then in carnivores (Luskin and Shatz, 1985) (for an interesting review on the history of research on the SP, see Judas et al. (2010). The SP was defined as a transient layer located above the intermediate zone and below the cortical plate (sub-plate) in the developing cerebral cortex. The cortical plate later forms the neocortical layers 2–6 (for review, see Bystron et al., 2008). During mammalian evolution the SP became profoundly thicker and in human prenatal neocortex the SP is between 1.5 and 4 times thicker than the cortical plate (Figures 1A,B; Vasung et al., 2016; for review, see Judas et al., 2013; Hoerder-Suabedissen and Molnár, 2015). The SP is a transient structure being present only during early corticogenesis. However, depending on the mammalian species SP neurons (SPNs) may survive under physiological or pathophysiological conditions into adulthood.

Over the last 40 years we gained more and more knowledge on the functional role of SPNs in neocortical development. The SP is the first cortical site receiving synaptic inputs from the thalamus, other cortical regions and from neuromodulatory nuclei (for review, see Kanold and Luhmann, 2010). It has been initially suggested that the SP serves as a transient “waiting zone” for the ingrowing thalamocortical axons (for review, see Allendoerfer and Shatz, 1994). Subsequently it became clear that the SP is more than a passive waiting station for ingrowing thalamocortical fibers and a number of experimental studies in different species demonstrated that the SP is an essential element in early cortical function and development, being critically involved in the formation of cortical columnar structure, the maturation of intracortical inhibition, and the occurrence of ocular dominance plasticity (for review, see Friedlander and Torres-Reveron, 2009; Luhmann et al., 2009; Kanold and Luhmann, 2010; Kostovic and Judas, 2010; Hoerder-Suabedissen and Molnár, 2015).

While various aspects of the SP function have been covered by previous review articles, recent reports provided additional information on the molecular diversity and fate of SPNs, revealed additional roles of SPNs in neocortical development (e.g., neuronal migration), and provided evidence for an involvement/alteration of SPNs in brain diseases. Therefore, this review article aims (i) to give a comprehensive overview of our current understanding on the origin, molecular heterogeneity, physiological properties and synaptic integration of SPNs, (ii) to integrate recent findings into a concept of the functional role of SPNs in the structural and functional maturation of the cerebral cortex, and (iii) to present hypotheses how impaired SP function may contribute to the etiology of neurological or neuropsychiatric disorders.

## Morphological and Molecular Properties

The SP contains in- and outgrowing axons, glial cells as well as migratory and postmigratory neurons, but only the postmigratory neurons in this layer are termed SPNs (Kostovic and Rakic, 1990). Histological and molecular studies have shown that SPNs consist of a variety of morphologically and biochemically distinct subpopulations. Early studies also demonstrated a large morphological variety of SPNs (Allendoerfer and Shatz, 1994). SPNs encompass pyramidal-like and inverted pyramidal-like (main dendrite with ventricular orientation) neurons, horizontally oriented neurons, neurogliaform cells as well as polymorphic neurons (Valverde et al., 1989; Kostovic and Rakic, 1990; Hanganu et al., 2002; Hoerder-Suabedissen and Molnár, 2012). Both, smooth and spiny dendrites have been described for SPNs (Mrzljak et al., 1988; Valverde et al., 1989; Hanganu et al., 2002). Because of the high morphological resemblance of SPNs to layer 6B (L6B) neurons, it has been recently suggested that L6B consists of persistent non-pyramidal neurons from the SP and neocortical L6B pyramidal neurons (Marx et al., 2017). Axonal collaterals of SPNs show a dense arborisation within the SP, but also project to the cortical plate and up to the marginal zone (Friauf et al., 1990; Clancy and Cauller, 1999; Myakhar et al., 2011). The intracortical axonal arborisation is, however, dynamic and influenced by sensory experience, as demonstrated in a subset of SPNs in the Golli-tau model (Pinon et al., 2009). In addition to intracortical targets, axons of SPNs also extend to subcortical regions (McConnell et al., 1989, 1994; De Carlos and O’Leary, 1992; Hoerder-Suabedissen and Molnár, 2012). In general, the somatic morphological appearance of SPNs could not be correlated with their axonal arborization pattern, with the exception that inversed pyramidal neurons seem to have only intracortical axonal projections (Hoerder-Suabedissen and Molnár, 2012).

The morphological heterogeneity of SPNs is accompanied by a variability in their molecular properties. Immunohistochemical studies, investigating the presence of GABA, GABA synthesizing enzymes or vesicular transporters, demonstrated that both GABAergic and glutamatergic neurons can be found in the SP (Chun et al., 1987; Wahle et al., 1987; Finney et al., 1998; Del Rio et al., 2000; Ina et al., 2007; Hackett et al., 2016). Recently it has been demonstrated that three persistent subpopulations of GABAergic SPNs could be separated by their exclusive expression of somatostatin (30% of total GABAergic SPNs), 5HT_3A_ serotonin receptors (60%), and parvalbumin (10%) (Qu et al., 2016). The somatostatin subgroup coexpresses neuronal nitric oxide synthase (nNOS), calbindin (CB) and neuropeptide Y (NPY). The 5HT_3A_ subgroup coexpresses calretinin (CR) and the GABA_A_ receptor subunit delta, which is also coexpressed in the PV subgroup (Qu et al., 2016). At least a fraction of the GABAergic SPNs survive into adulthood and probably establish long-ranging GABAergic connections (Clancy et al., 2009). Although some studies indicate that dopaminergic neurons may exist in the SP, a recent report documents that the expression of dopa-decarboxylase in the mouse SP is limited to non-neuronal cells only (Hoerder-Suabedissen et al., 2009).

Several markers have been suggested to enable selective labeling of SPNs (see e.g., Table 2 in Kanold and Luhmann, 2010), but many of them are expressed initially only in the SP with a delayed expression in other cortical layers (Hoerder-Suabedissen et al., 2013) or are exclusively found in the SP at prenatal stages (Oeschger et al., 2012). Early attempts to find subtype specific markers also identified a monoclonal antibody against immunoglobulin motifs (SP1) that specifically labels SPNs in cats (Henschel and Wahle, 1994). However, which proteins express these immunoglobulin motifs in the SP is unknown and the specificity of this antibody in other species has not been further investigated. The neuropeptides somatostatin and NPY are present at perinatal stages and almost selectively expressed in SPNs (Robertson et al., 2000), while at later postnatal stages these neuropeptides are also expressed in other neurons. Similar findings have been made for progesterone receptors, which appear in rat SPNs at E18, while in more superficial layers their expression is delayed by several days (López and Wagner, 2009). Neurons expressing acetylcholine esterase are also enriched, but not exclusively located, in the SP (Robertson et al., 2000). In rodents kynurein aminotransferase is exclusively expressed in the SP during the first postnatal week, but only in a small subpopulation of SPNs (Csillik et al., 2002). Neurotrophin receptors have been documented in the human SP already in 1990 (Allendoerfer et al., 1990) and the neurotrophin receptor p75NTR has been subsequently been found in the rodent SP between E16 and P7 (McQuillen et al., 2002). In contrast the cortical plate and superficial cortical layers completely lack neurotrophin receptors with the exception of Cajal-Retzius neurons in the marginal zone (McQuillen et al., 2002; Blanquie et al., 2017b). However, no information is currently available whether all or just subpopulations of SPNs are positive for p75NTR.

Using expression profiling Hoerder-Suabedissen and coworkers identified an increased expression in a variety of genes in the mouse SP. However, only 68 of these genes show a SP-specific expression at least at a defined developmental stage (Hoerder-Suabedissen et al., 2013). Only seven of these genes are specifically expressed in the SP throughout development and may be useful as reliable, specific markers for SPNs. In particular, these genes are Nurr1, Ctgf, Cplx3, Nxph4, Inpp4b, Htr1d, and Tpd52l1 (see Table 1), with Nurr1, however, also labeling supragranular layers in lateral regions of the neocortex (Hoerder-Suabedissen et al., 2009, 2013). For Nurr1, Cplx3, CTGF and Tpd52l1 the translation of these genes into SP-specific proteins has also been demonstrated (Hoerder-Suabedissen et al., 2009, 2013) (Figures 1C,D). At least the markers Nurr1, Clpx3, Lpar1 and CTGF demonstrate a fairly overlapping expression in mice (Hoerder-Suabedissen and Molnár, 2013), indicating that they do not represent markers for molecular distinct subpopulations. However, as Nurr1 and Cplx3 are virtually absent in GABAergic SP cells, these markers most probably label a subpopulation of glutamatergic neurons (Hoerder-Suabedissen and Molnár, 2013). Nurr1 and CTGF have also been found in the SP of humans (Wang et al., 2010), indicating that the human SP shows some similarity in the gene expression pattern to the rodent SP. Interestingly, a significant fraction of SP specific genes has been associated to neurodevelopmental disorders, such as autism spectrum disorders and schizophrenia (Hoerder-Suabedissen et al., 2013).

**Table 1:** SP-specific genes and their proposed cellular functions.

Gene	Full Name	Function	Reference
Nxph4	Neurexophilin 4	Unknown	
Nurr1	Nuclear receptor related 1	Orphan nuclear hormone receptor	Arimatsu et al., 2003
Cplx3	Complexin 3	Vesicular release	Hoerder-Suabedissen et al., 2013; Mortensen et al., 2016
Inpp4b	Inositol phosphate 4 phospatase II	Phosphatidylinositol signaling	Agoulnik et al., 2011; Hoerder-Suabedissen et al., 2013
Ctgf	Connective tissue growth factor	Growth factor	Heuer et al., 2003
Htr1d	5HT 1D receptor	Metabotropic serotonin receptor	Hoerder-Suabedissen et al., 2013
Tpd52l1	Tumor protein D52-like 1	Lipid metabolism	Hoerder-Suabedissen et al., 2013; Kamili et al., 2015

In summary, these studies provide a set of molecular markers that are fairly specific for the SP during early developmental stages, but are most probably expressed only in subpopulations of SP neurons. Until now we lack a common molecular marker that unequivocally identifies all SPNs, emphasizing that the SP consists of a set of neuronal subpopulations with different molecular properties, distinct functions and diverse ontogenetic origins (Hoerder-Suabedissen and Molnár, 2015). On the other hand, no clear correlations between the expression profile of molecular markers and morphological patterns of SPNs have been obtained yet.

## Developmental Origin of SPNs

Birthdating studies revealed that most SPNs are generated between E11 and E13 in the mouse and between E13 and E15 in the rat (Wood et al., 1992; Valverde et al., 1995a; Price et al., 1997; Hoerder-Suabedissen and Molnár, 2013), thus belonging to the earliest generated neocortical neurons. Data obtained in rodents indicate that the SP in medial parts of the neocortex is populated by slightly earlier born (E12.5) neurons (Hoerder-Suabedissen and Molnár, 2013). In contrast, in primate neocortex the majority of SPNs are generated before the cortical plate has been formed and the generation of the SP continues until around mid-gestation (Molnár et al., 2006). In the primate fetal cortex, SPNs initially form a dense band, comparable to what has been shown in rodents (Figures 1A,B), which subsequently becomes dispersed through the thickening of the SP (Duque et al., 2016). It has been suggested that the invasion of monoamine, basal forebrain, thalamocortical and corticocortical axons may regulate this region-dependent dispersion of SPNs (Duque et al., 2016).

The majority of early born SPNs are generated in the ventricular zone. These neurons initially populate the preplate, which later is separated by subsequently born and outwardly migrating neurons into the superficial marginal zone (MZ) and the basal SP (Bystron et al., 2008). Recently a subpopulation of early born SPNs have been identified, which are generated in the rostromedial telencephalic wall, migrate tangentially to populate the preplate, and express the SP markers CTFG, Cplx3 or Nurr1 (Pedraza et al., 2014). Furthermore, GABAergic SPNs originate in the medial ganglionic eminence (Lavdas et al., 1999), the most important ontogenetic source of neocortical GABAergic neurons (Anderson et al., 1999), and reach the developing neocortex via tangential migration (Parnavelas, 2000).

To address the phylogenetic origin of the SP, Zoltan Molnár and coworkers used a set of SP specific markers, as identified from the murine SP, and studied their expression in the developing brain of several species (Wang et al., 2011). Interestingly, Moxd1, Ddc and Trh, all typical markers of mouse SPNs, are absent in the rat SP, illustrating a high variability of gene expression already at closely related species (Wang et al., 2011). In the short tailed opossum the SP markers Ctfg, Nurr1 and Moxd1 are expressed intermingled in neurons at the lower part of the developing cortex, suggesting that these neurons may represent the homologue of the SP in the marsupial brain. Expression of Ctfg and Nurr1 has also been found in the developing turtle brain in the so-called cell dense layer, which is probably homologous to the lower part of the neocortex. Moreover, in the pigeon brain Ctgf, Moxd1, and Nurr1 are expressed mainly in the developing hyperpallium, which probably represents a functional homologue of the neocortex. Thus neurons with molecular and functional properties corresponding to SPNs may have emerged early during amniote evolution (Wang et al., 2011). However, it has been suggested that the SP of mammals incorporates both, neuronal subpopulations that are homologous to sauropsidian ancestors as well as subpopulations that appeared later during mammal evolution (Montiel et al., 2011; Judas et al., 2013). This hypotheses is supported by the observation that the expression profile of SP specific genes in the human fetal brain is different from the rodent profile (Miller et al., 2014), which illustrates that the evolutionary achievement of the complex neocortex in humans is not only correlated to a thicker SP (Judas et al., 2013; Kostovic et al., 2014), but most probably by the addition of new molecular/functional distinct SPN populations.

## Intrinsic Membrane Properties

**FIGURE 2:**
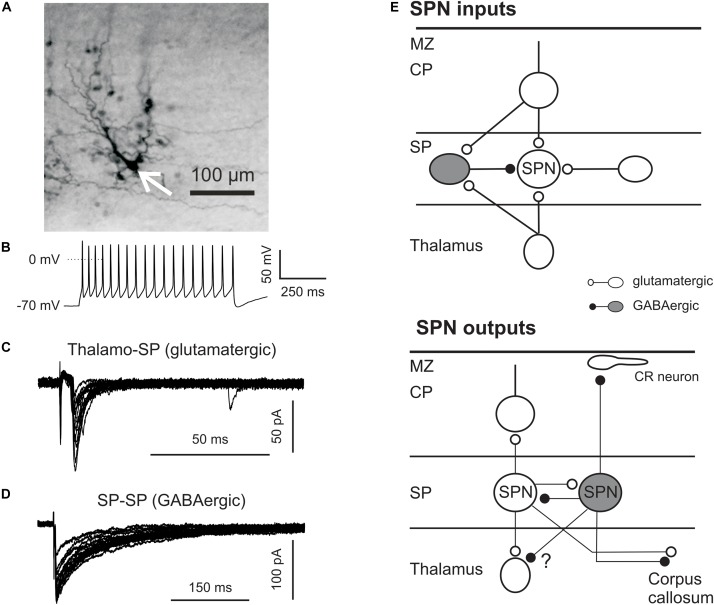
Synaptic connectivity of SPNs. **(A)** Intracellular biocytin staining of one SPN (arrow) in P3 mouse neocortex reveals columnar arrangement of several dye-coupled cells (with permission from Dupont et al., 2006). **(B)** Representative firing pattern induced by suprathreshold depolarization of SPN in P2 mouse cortex. SPNs were identified by their location, their appearance in differential interference contrast videomicroscopic image, and their electrophysiological properties. **(C)** Glutamatergic PSCs elicited by electrical stimulation in the thalamus. **(D)** GABAergic PSCs induced by electrical stimulation of the SP. **(E)** Schematic drawing illustrating SPN inputs (top) and outputs (bottom). CP, cortical plate; CR, Cajal-Retzius neuron in the marginal zone (MZ).

Despite their diverse origins and large variety of morphological and molecular features, SPNs display rather similar electrophysiological properties. SPNs have a resting membrane potential of about -55 mV. Their membrane resistance is higher than 1 GΩ, indicating that even small postsynaptic currents in sparsely connected immature networks may be able to elicit action potentials (APs) in SPNs (Luhmann et al., 2000; Hanganu et al., 2001; Zhao et al., 2009; Liao and Lee, 2012; Unichenko et al., 2015). On the other hand the slow membrane time constant and low resonance frequency of SPNs (Sun et al., 2012) support the summation of recurrent subthreshold synaptic inputs occurring, e.g., during thalamic network bursts (Yang et al., 2013). In the prenatal rodent cortex most SPNs respond to sustained depolarizing current injection with overshooting APs and repetitive firing (Figure 2B). SPNs revealed APs with more mature properties, i.e., faster kinetics and larger amplitudes, as compared to APs of immature cortical neurons located in the marginal zone or in the cortical plate, due to the fact that they show largest amplitudes of voltage-dependent sodium currents (I_Na_) (Luhmann et al., 2000), for review (see Luhmann et al., 2009; Kanold and Luhmann, 2010). Mean firing rate is about 10–20 Hz, but some SPNs can fire APs at frequencies up to 40 Hz (Hanganu et al., 2001; Unichenko et al., 2015). In the mouse auditory cortex SPNs appear to be capable of repetitive firing only from P5 on (Zhao et al., 2009). Similar properties of SPNs were observed in postmortem human fetal brain tissue within the second trimester of gestation (16–23 gestation weeks). Here, resting membrane potential is about -55 mV and input resistance amounts to ∼2 GΩ. Human SPNs display large I_Na_ (>500 pA) and are capable of firing repetitive APs in response to sustained depolarization (mean frequency ∼20 Hz) (Moore et al., 2009). Compared to other neurons in the developing neocortex, the mature APs of SPNs thus enable effective transmission of neuronal activity to cortical and subcortical target cells by means of AP burst firing.

Interestingly, SPNs *in vitro* are spontaneously active in the immature human brain (16–23 gestation weeks) and can generate activity patterns similar to UP- and DOWN-states in adult rodent cortex. The typical shape of UP-states is a relatively long plateau depolarization with overshooting APs. Mean duration of Up-state amounts to ∼900 ms, but their occurrence do not show any temporal regularity (Moore et al., 2011). Although the density of connexin hemichannels in the SP is high, human SPNs appear to be only weakly coupled via gap junctions. However, spontaneous opening of connexin hemichannels leads to SPN depolarization and seems to underlie the spontaneous neuronal activity in the SP (Moore et al., 2014). In contrast to the human SP, SPNs in rodents demonstrate a strong gap-junction coupling not only between SPNs but also between SPNs and neurons of the cortical plate (Figure 2A) (Dupont et al., 2006). Rodent SPNs in acute brain slices display low level of spontaneous activity, but neuronal activity similar to that observed in human SPNs can be induced by activation of acetylcholine receptors (Dupont et al., 2006; Hanganu et al., 2009). Spontaneous neuronal activity of SPNs in combination with strong gap junction-mediated coupling between SPNs and cortical plate neurons may provide a prerequisite for the activity-dependent formation of the columnar organization of the cortex before coherent sensory information is transmitted from subcortical sources.

## Synaptic Connectivity (Inputs and Outputs)

Results obtained by different experimental techniques and in various species confirm the hypothesis that SPNs are functionally well integrated in the developing cerebral cortex. SPNs play a central role in the establishment and functional maturation of thalamocortical connections and project to the growing cortical plate projections (McConnell et al., 1989; Ghosh et al., 1990). Ultrastructural studies of fixed brains revealed both symmetrical and asymetrical synapses in the SP, suggesting that SPNs receive both glutamatergic and GABAergic inputs (Kostovic and Rakic, 1990; Herrmann et al., 1994). In the visual cortex of perinatal kittens SPNs receive excitatory inputs via axons traversing the developing white matter (Friauf et al., 1990; Friauf and Shatz, 1991). In mice thalamocortical axons reach the SP at E15 and 1 day later begin to invade the upper cortical layers (Del Rio et al., 2000). *In vivo* electrophysiological recordings performed in young ferrets directly demonstrate that sound-evoked response in the auditory cortex emerge first in SPNs (Wess et al., 2017). Synaptic inputs to SPNs were investigated in detail in early postnatal neocortical brain slices of somatosensory and auditory cortex using local electrical and optical stimulation. Electrical activation of thalamocortical fibers reliably elicits AMPA and NMDA receptor-mediated excitatory postsynaptic currents (EPSCs) in SPNs already at P0 (Hanganu et al., 2002; Zhao et al., 2009). Similar results were obtained in prenatal rat slices using voltage-sensitive dye imaging (Higashi et al., 2002) and current source-density analyses in perinatal neocortical rat slices (Molnár et al., 2003), demonstrating a functional glutamatergic input from the thalamus (Figure 2C). In addition to thalamocortical inputs, SPNs also receive glutamatergic inputs from glutamatergic neurons in the SP (Hanganu et al., 2002; Zhao et al., 2009). Comparison of thalamocortical and intra-SP glutamatergic inputs in somatosensory cortex revealed significant differences in their short-termed synaptic plasticity (Hirsch and Luhmann, 2008). Intra-SP evoked EPSCs reveal strong paired-pulse facilitation, while thalamocortically evoked EPSCs demonstrate a slight paired-pulse depression, suggesting that the presynaptic release probability is higher at thalamocortical synapses as compared to intra-SP inputs. Indeed thalamocortically evoked EPSCs have larger amplitudes but they are exhausted already at relatively low (<40 Hz) stimulation frequencies, while intra-SP connections are capable to support information transfer even at 100 Hz (Hirsch and Luhmann, 2008). The last observation in combination with the strong gap junction-mediated coupling of SPNs favor the hypothesis that the SP during perinatal period functions as an amplifier of incoming thalamocortical activity (for review, see Kanold and Luhmann, 2010). A third excitatory synaptic input originates from the developing cortical plate. Both electrical and optical stimulation (using the glutamate uncaging approach) in the cortical plate elicits EPSCs both in somatosensory and auditory SPNs (Hanganu et al., 2002; Zhao et al., 2009). Interestingly in the auditory cortex at least some of these connections appear to be postsynaptically silent during the first postnatal week (Meng et al., 2014). In addition to these glutamatergic inputs, SPNs receive GABAergic inputs from neighboring GABAergic SPNs (Figure 2D) (Luhmann et al., 2009; Unichenko et al., 2015). Paired-pulse stimulation of GABAergic fibers reveal strong paired-pulse depression and a relatively high presynaptic release probability, suggesting that GABAergic SPNs can effectively control excitability and information transfer within the SP. It has been shown that the GABA transporter GAT-2/3 in SPNs operates in reverse mode and that GABA released via GAT-2/3 activates presynaptic GABA-B receptors on GABAergic synapses and tonically inhibits GABAergic inputs on SPNs (Unichenko et al., 2015). SPNs probably also receive corticocortical long-distance synaptic inputs, for example from callosal projections (Ivy and Killackey, 1981), but the functional properties of these projections are not characterized yet (Figure 2E). Using the *in vitro* photostimulation approach Patrick Kanold and coworkers identified in mouse auditory cortex two classes of SPNs with distinct spatial patterns of glutamatergic and GABAergic synaptic inputs (Viswanathan et al., 2012). The first class of SPNs receives inputs from only deep neocortical layers. The second class of SPNs is innervated from deep as well as superficial layers including layer 4 and is located more superficially in the SP as compared to the other class of SPNs.

In addition to glutamate and GABA, other neurotransmitter systems also play an important role in the SP. SPNs express both functional nicotinic (Hanganu and Luhmann, 2004) and muscarinic acetylcholine receptors of m1/m5 subtype (Hanganu et al., 2009). The cholinergic modulation of SPNs plays an essential role in the activation of oscillatory network events in the superficial cortical plate of immature rodents (Dupont et al., 2006). Similarly SPN excitability can be modulated by ambient glycine and/or taurine via activation of glycine receptors (Kilb et al., 2008; Kilb, 2017; Kilb and Fukuda, 2017). In the mouse prefrontal cortex, SPN activity can be induced via activation of purinoreceptors, which *in situ* seems to occur by ATP release from astrocytes, indicating that astrocytes may modulate early neuronal activity in the SP (Beamer et al., 2017). Serotonin appears not to influence intrinsic properties of SPNs, but it presynaptically suppresses thalamo-SP afferents through 5HT_1B_ receptors (Liao and Lee, 2014).

Subplate neurons not only contact local cells but also form long-distance projections. Using carbocyanine dye tracing three groups of SPNs with different projection targets have been demonstrated in the SP of the mouse somatosensory cortex during the first postnatal week (Hoerder-Suabedissen and Molnár, 2012). The first group of SPNs projects into the developing somatosensory cortex. At P2 their axons reach the marginal zone, while at P7 they end at or below layer 4. The existence of SPN projections to the marginal zone has been shown anatomically in kitten visual cortex (Friauf et al., 1990) and functionally in mouse somatosensory cortex (Myakhar et al., 2011). In mouse auditory cortex glutamate uncaging in the SP elicit an excitatory response in layer 4 neurons, confirming direct synaptic inputs from the SP (Zhao et al., 2009). The second SPN group projects via the internal capsule to the dorsal thalamus mediating a corticothalamic feedback loop. In the embryonic brains of the Golli-tau-eGFP mouse the growth of these projections has been shown to be interrupted by a transient pause before invading the thalamus (Jacobs et al., 2007). The third group of SPNs seems to project to the contralateral hemisphere. These SPNs can be back-labeled from dye placement in the corpus callosum or in the contralateral site of the stained cells. The existence of callosally projecting SPNs has been previously documented in cat and ferret visual cortex (Antonini and Shatz, 1990) and confirmed in embryonic Golli-tau-eGFP mice (Jacobs et al., 2007). SPN projections to the superior colliculus has been also reported in mice (Jacobs et al., 2007) and in cats and ferrets (Antonini and Shatz, 1990).

Interestingly there seems to be a tendency that glutamatergic SPNs projecting toward neocortical layers reside preferentially in the superficial SP (Finney et al., 1998), suggesting the existence of functional sublayers in the SP. The recent identification of genetic markers for SPN subpopulations (see above) allows the detailed characterization of the specific projection patterns (Hoerder-Suabedissen et al., 2018). It has been demonstrated that Cplx3 positive SPNs preferentially extend their axons to septal regions of the somatosensory cortex as well as to the medial posterior nucleus of the thalamus, suggesting that they are preferentially involved in paralemniscal pathways (Viswanathan et al., 2017). These results suggest that some SPNs can link thalamocortical and corticothalamic neuronal circuits both during early postnatal development and in adulthood (Figure 2E). New reporter mouse lines will help to further characterize the connectivity of distinct SPNs, while application of optogenetics will allow to unravel the development and function of SPN inputs and outputs during perinatal development.

## Function in Early Network Activity

As discussed in the previous sections, SPNs are electrophysiologically characterized by their relative mature intrinsic membrane properties (e.g., firing pattern), their pronounced connectivity via chemical and electrical synapses, and their synaptic activation by neuromodulatory inputs (e.g., cholinergic). These functional properties place SPNs in an ideal position to serve as key elements in monitoring and controlling neuronal network activity during a critical period of early cortical development (for review, see Luhmann et al., 2009; Kanold and Luhmann, 2010; Colonnese and Phillips, 2018). It is therefore not surprising that SPNs play an important role in early neocortical activity, although they probably not generate, but rather transmit and amplify the activity patterns. The cerebral cortex of newborn rodents shows two distinct patterns of spontaneous and sensory evoked activity. (i) Spindle bursts with a frequency of 10–25 Hz, a duration of 0.5–3 s and occurring spontaneously every ∼10 s (for review, see Yang et al., 2016; Luhmann, 2017). (ii) Early gamma oscillations with a frequency of 30–50 Hz and a duration of 100–300 ms (for review, see Khazipov et al., 2013; Luhmann and Khazipov, 2017). Extracellular multi-electrode recordings in newborn rat barrel cortex *in vivo* demonstrated that the current sink of this early activity is located in the inner layer of the cortical plate (future layer 4) and in the SP (Yang et al., 2009; Mitrukhina et al., 2015). Spindle burst (delta brush) activity has been also recorded with advanced EEG recording techniques in preterm human babies at a developmental stage when the human neocortex resembles that of the newborn rodent (Milh et al., 2007; Tolonen et al., 2007; Stjerna et al., 2012) (for a remarkable demonstration of EEG activity in preterm human cortex and the role of the SP see https://www.jove.com/video/3774/preterm-eeg-a-multimodal-neurophysiological-protocol).

Interestingly, these early activity patterns are present in somatosensory (Khazipov et al., 2004; Minlebaev et al., 2011; Yang et al., 2013), visual (Hanganu et al., 2006), auditory (Chipaux et al., 2013) and motor (An et al., 2014) cortex and either occur spontaneously or can be elicited by mild sensory stimulation (for review, see Colonnese and Khazipov, 2012; Luhmann and Khazipov, 2017). Furthermore, they are modulated by the cholinergic system (Hanganu et al., 2007) acting on SPNs (Hanganu et al., 2009). Therefore oscillatory network activity in the frequency range of spindle bursts and gamma oscillations can be also elicited in thick neocortical slices or intact *in vitro* preparations of a whole cortical hemisphere by application of cholinergic (muscarinic) agonists, such as carbachol, and require an intact SP (Dupont et al., 2006). These *in vitro* observations are supported by *in vivo* experiments, which demonstrate that endogenous and sensory evoked spindle burst activity in the neonatal rat somatosensory cortex is largely abolished after removal of the SP (Tolner et al., 2012).

## Role in Early Cortical Development

The seminal work of Carla Shatz and coworkers demonstrated in the visual system of cats and ferrets that the SP plays a pivotal role in the development of the thalamocortical connection (Shatz and Luskin, 1986; Friauf et al., 1990; Ghosh et al., 1990), the feedback projection from the SP to the thalamus (McConnell et al., 1989; McConnell et al., 1994), and the activity-dependent development of cortical columns and GABAergic inhibition (Ghosh and Shatz, 1992; Lein et al., 1999; Kanold et al., 2003; Kanold and Shatz, 2006). Recent evidence obtained in mouse auditory cortex indicates that SPNs regulate the maturation of intracortical inhibition by direct and indirect SP synaptic inputs to GABAergic interneurons (Deng et al., 2017).

In newborn (P0-P1) rodent cortex, spontaneous or sensory evoked activation of SPNs elicits a spatially confined spindle burst or gamma oscillation, which synchronizes a local columnar network via neuronal, connexin-36 containing gap junctions (Dupont et al., 2006; Hanganu et al., 2009). This SP-driven activity probably represents the functional blueprint for the development of columnar networks (Yang et al., 2013), since selective lesioning of the SP eliminates spindle burst activity and prevents the development of modular architecture in the barrel cortex (Tolner et al., 2012).

More recently another important role of SPNs has been demonstrated in mouse embryonic cerebral cortex, namely the control of radial neuronal migration (Ohtaka-Maruyama et al., 2018). SPNs form transient glutamatergic synapses with migrating excitatory neurons just below the SP and facilitate their multipolar-to-bipolar transition by NMDA receptor-mediated synaptic transmission. This causes a change in the migration mode from slow multipolar migration to faster radial glial-guided locomotion (Ohtaka-Maruyama et al., 2018). An involvement of the SP in the control of late neuronal migration and the potential relevance for pathogenesis of neuronal migration disorders has been previously proposed by Kostovic et al. (2015). It remains to be studied in more detail whether and to which extent neurological (e.g., epilepsy) and neuropsychiatric diseases (e.g., schizophrenia, autism spectrum disorder) may be related to a dysfunction of the SP in regulating this step in neuronal migration (see section below).

## Developmental Destiny of SPNs

The SP has been considered as a transient structure serving a transient function. However, species-dependent differences in the amount of death versus survival of SPNs have been documented (for review, see Friedlander and Torres-Reveron, 2009; Kanold and Luhmann, 2010; Hoerder-Suabedissen and Molnár, 2015). Kostovic and Rakic demonstrated in human and monkey cerebral cortex already four decades ago that so-called interstitial cells in the white matter (WM) remain in the adult brain and suggested that these ”interstitial cells represent a vestige of the transient embryonic subplate layer” (Kostovic and Rakic, 1980). Meanwhile there is compelling evidence that SPNs, identified by their early generation, genetic markers and/or morphological properties, persist into adulthood, either as WM neurons (Valverde and Facal-Valverde, 1988) or forming a dense band below layer 6, which is termed either layer 6b, layer 7 or subgriseal layer (Chun and Shatz, 1989; Valverde et al., 1989; Woo et al., 1991; Reep, 2000; Marx and Feldmeyer, 2013; Marx et al., 2017). In rodents many Clpx3-positive glutamatergic SPNs persist in layer 6b of the adult neocortex (Viswanathan et al., 2017), while virtually all somatostatin-positive and NPY-positive SPNs disappear between P7 and P14 (Arias et al., 2002). These observations suggest that subpopulations of SPNs most probably have different development fates.

It has been estimated that only 10–20% of SPNs persist until adulthood (Friedlander and Torres-Reveron, 2009), although some studies assume in rodents a higher portion of surviving SPNs (Woo et al., 1991; Valverde et al., 1995b; Robertson et al., 2000). Clearly the majority of SPNs must disappear during early development by programmed cell death. In rodents most SPNs disappear during the first postnatal week and the rate of cell death is reduced in subsequent stages (Price et al., 1997). Interestingly the fraction of SPN loss depends critically on their birthday. While virtually all SPNs born at E11 disappear during the first postnatal week, a substantial fraction of E12 and E13 born SPNs persists until at least P21 (Price et al., 1997). These surviving SPNs persist in the WM and layer 6B, show mature electrophysiological properties and are integrated into the synaptic network via glutamate and GABA receptors (Torres-Reveron and Friedlander, 2007).

Subplate neurons disappear by programmed cell death, as apoptotic neurons have been identified in the SP by TUNEL assay, electron microscopy or the appearance of pyknotic nuclei (Spreafico et al., 1995; Rakic and Zecevic, 2000; Robertson et al., 2000). In the postnatal rodent neocortex an obvious increase of apoptotic cells can be observed in the SP between P5 and P8, when apoptotic SPNs outnumber apoptotic cells in all other layers (Spreafico et al., 1995), but see Valverde et al. (1995b), thus supporting the observation that a substantial fraction of SPNs disappear during early postnatal development. Different mechanisms have been suggested that drive SPNs into apoptosis. The down regulation of kynurenic aminotransferase, an enzyme that catalyzes the formation of the endogenous NMDA receptor blocker kynurenic acid, in SPNs after P7 has been suggested to boost excitotoxicity in SPNs (Csillik et al., 2002). In addition, the down regulation of the neurotrophin receptor p75NTR after P7 (McQuillen et al., 2002) may also trigger cell death. In SPNs this receptor supports neuronal survival (DeFreitas et al., 2001), in contrast to its classical contribution in proapoptotic signaling in most other cells (Nykjaer et al., 2005). Finally, the elimination of thalamic inputs during functional maturation of the neocortex (Friauf and Shatz, 1991; Kanold et al., 2003) deplete SPNs from their essential trophic support by these axons (Price and Lotto, 1996). This synaptic deprivation may also reduce the activity of SPNs, which can trigger neuronal apoptosis (for review, see Blanquie et al., 2017a).

## Patho(Physio)Logy of SPNs

A central role of the SP in structural and functional plasticity of the human cerebral cortex after perinatal brain damage has been proposed three decades ago by Kostovic et al. (1989). It has been further suggested that SPNs and early neocortical circuits may be preserved following genetic or pathophysiological disturbances causing long-term neurological and psychiatric disorders (for review, see Luhmann et al., 2003). This hypothesis is of clinical relevance since in human early (preterm) development hypoxia-ischemia or infection may cause damage to the WM and the SP leading to periventricular leukomalacia (PVL). Transient hypoxia-ischemia in the neonatal rat reveals a selective vulnerability of SPNs and reproduces the WM injury and deficits in motor function observed in human PVL (McQuillen et al., 2003). This *in vivo* study is supported by *in vitro* data demonstrating a higher susceptibility of SPNs to oxygen-glucose deprivation when compared to other neocortical neurons, which may result from differences in the expression of glutamate receptors (Nguyen and McQuillen, 2010). In particular calcium-permeable AMPA receptors may contribute to this selective vulnerability (Hsu et al., 2010). Combined oxygen and glucose deprivation *in vitro* causes a prominent ischemic membrane depolarization in SPNs, which can be significantly delayed and reduced by blockade of NMDA receptors with MK-801 (Albrecht et al., 2005). However, the question whether SPNs reveal a selective vulnerability to hypoxia-ischemia is controversial (for review, see Millar et al., 2017). A recent *in vivo* study using the neonatal rat model of hypoxia-ischemia could not confirm previous reports (Okusa et al., 2014). In the fetal sheep SPNs even show a prominent resistance to early hypoxia-ischemia and survive, but dendritic arborization and functional maturation of SPNs is impaired (McClendon et al., 2017). Another approach addressing changes in the synaptic connectivity of SPNs following a mild or a more severe hypoxic-ischemic insult in newborn rats has been used by Patrick Kanold and coworkers (Sheikh et al., 2018). Histological damage to the SP could be observed only in the severe model. However *in vitro* laser-scanning photostimulation revealed a hyperconnectivity of excitatory and inhibitory synaptic inputs onto SPNs in both models, indicating that immature circuits involving SPNs are highly susceptible to early hypoxia-ischemia. In line with this, hypoxia-ischemia in newborn rats causes a reduction in spindle burst activity, deficits in dendrite and spine formation in neocortical pyramidal neurons and a delayed expression of glutamate receptor subunits and transporters (Ranasinghe et al., 2015). These hypoxia-ischemia induced modifications in synaptic connectivity to SPNs may contribute to an impairment of activity-dependent synaptic plasticity during further development (Failor et al., 2010).

On the other hand, altered connectivity is a key observation in patients with neuropsychiatric disorders, including autistic spectrum disorder and schizophrenia (Kostovic et al., 2010; Itahashi et al., 2014; Kambeitz et al., 2016), indicating that dysfunction of SPNs during development may be involved in the etiology of such disorders. In line with this, using a pharmacological animal model of autism spectrum disorder (prenatal valproic acid exposure) Kanold and colleagues demonstrated in the mouse auditory cortex *in vitro* an increased excitatory and inhibitory connection probability or strength during the first postnatal week, which during this period critically depends on SPNs, which progress into a general hyperconnectivity after P10 (Nagode et al., 2017). Finally, an increased number of interstitial cells has been reported in patients with schizophrenia (Eastwood and Harrison, 2005), indicating that an enhanced number of surviving SPNs may also be involved in the etiology of this disease. These data further support the hypothesis that SPNs do not only fulfill an important role in the physiological development of the neocortex, but also indicate that disturbances in the activity of SPNs may contribute to the manifestation of long-term neurological or psychiatric disorders (for review, see Hutsler and Casanova, 2016; Hadders-Algra, 2018).

In recent years the role of SPNs in neocortical development of humans suffering from pre- or neonatal hypoxia-ischemia has been studied to some extent. Preterm infants with PVL show neuronal cell loss in the SP and WM indicating a vulnerability of these neurons in humans (Kinney et al., 2012). This conclusion is supported by immunohistochemical studies in post-mortem neocortical tissue from very preterm and preterm infants (postconceptional week 24–29 and 30–34, respectively) with periventricular white matter injury showing activated microglial cells in the SP (Pogledic et al., 2014). As discussed above, SPNs are required for spindle burst activity in the newborn rodent cortex. EEG recordings from premature infants with white matter and/or SP injury shows a depressed background activity and a loss of burst activity in the spindle frequency band (Ranasinghe et al., 2015), which is comparable to alterations in the spindle-burst activity observed in a rat model of prenatal hypoxia-ischemia observed in the same study.

Rodent studies have shown that the SP influences neuronal migration (see above). Therefore a similar function can be also postulated for the SP in primates (for review, see Kostovic et al., 2015). Migratory neurons may become misplaced within the SP resulting in migration disorders and cortical dysplasia. Neuronal migration disorders are often associated with pharmaco-resistant epilepsies (for review, see Guerrini et al., 2008). A large number of WM neurons, resembling SPNs, have been detected in surgically removed neocortical tissues of medically refractory temporal lobe epilepsy patients (Richter et al., 2016). Since all these patients were adult (up to 74 years of age) it can be assumed that SPNs survived in the cases. Neurosurgical removal of this focal cortical malformation generally results in a significant improvement or patients may become even seizure free, suggesting that the local neuronal circuits containing surviving SPNs are actually the epileptic focus (for review, see Luhmann et al., 2003). A similar hypothesis has been put forward by Kostovic and colleagues for the human prefrontal cortex (for review, see Kostovic et al., 2010). They propose that surviving SPNs and interstitial GABAergic neurons may cause an increased inhibition of prefrontal cortical neurons and circuits, which may be one mechanism underlying schizophrenia. These observations also indicate that structural and functional changes in the SP occur very early and clearly before the cerebral cortex has gained its typical six-layered architecture (for review, see Hutsler and Casanova, 2016).

## Conclusion and Future Perspectives

Over the last four decades we have gained a large amount experimental and clinical data demonstrating that SPNs play a fundamental role in early cortical development. For further understanding of the SP function we propose the following to-do-list:

(1) Classify different SPN populations on the basis of their molecular, morphological and electrophysiological properties.
(2) Identify the physiological, pathophysiological and genetic factors which control the death versus survival of SPNs or SPN subpopulations.
(3) Manipulate on a short-term (minutes to hours) and long-term (days to weeks) time-scale the function and activity of distinct SPN populations (e.g., by optogenetics) and analyze short- and long-term consequences.
(4) Characterize the functional role of SPNs in developing human cortex (e.g., in human brain organoids).
(5) Elucidate the transient and permanent role of SPNs in human neurological and psychiatric disorders.

## Author Contributions

All authors listed have made a substantial, direct and intellectual contribution to the work, and approved it for publication.

## Conflict of Interest Statement

The authors declare that the research was conducted in the absence of any commercial or financial relationships that could be construed as a potential conflict of interest.
